# Responsiveness and minimal important change of seven PROMIS computerized adaptive tests (CAT) in patients with advanced chronic kidney disease

**DOI:** 10.1186/s41687-023-00574-y

**Published:** 2023-04-04

**Authors:** Caroline B. Terwee, Esmee M. van der Willik, Fenna van Breda, Brigit C. van Jaarsveld, Marlon van de Putte, Isabelle W. Jetten, Friedo W. Dekker, Yvette Meuleman, Frans J. van Ittersum

**Affiliations:** 1 Department of Epidemiology and Data Science, Amsterdam UMC location Vrije Universiteit, P.O. box 7057, Amsterdam, 1007 MB the Netherlands; 2 Amsterdam Public Health research institute, Methodology, Amsterdam, The Netherlands; 3 Department of Clinical Epidemiology, Leiden University Medical Center, Leiden, The Netherlands; 4 Department of Nephrology, Amsterdam University Medical Centers, Amsterdam, The Netherlands

**Keywords:** Chronic kidney disease (CKD), Patient-reported outcomes measures (PROMs), Patient-reported outcomes Measurement Information System (PROMIS), Responsiveness, Minimal important change (MIC)

## Abstract

**Background:**

The Patient-Reported Outcomes Measurement Information System (PROMIS®) has the potential to harmonize the measurement of health-related quality of life (HRQL) across medical conditions. We evaluated responsiveness and minimal important change (MIC) of seven Dutch-Flemish PROMIS computerized adaptive tests (CAT) in Dutch patients with advanced chronic kidney disease (CKD).

**Methods:**

CKD patients (eGFR < 30 ml/min.1.73m^2^) completed at baseline and after 6 months seven PROMIS CATs (assessing physical function, pain interference, fatigue, sleep disturbance, anxiety, depression, and ability to participate in social roles and activities), Short Form Health Survey 12 (SF-12), PROMIS Pain Intensity single item, Dialysis Symptom Index (DSI), and Global Rating Scales (GRS) of change. Responsiveness was assessed by testing predefined hypotheses about expected correlations among measures, area under the ROC Curve, and effect sizes. MIC was determined with predictive modelling.

**Results:**

207 patients were included; 186 (90%) completed the follow-up. Most results were in accordance with expectations (70–91% of hypotheses confirmed), with some exceptions for PROMIS Anxiety and Ability to Participate (60% and 42% of hypotheses confirmed, respectively). For PROMIS Anxiety and Depression correlations with the GRS were too low (0.04 and 0.20, respectively) to calculate a MIC. MIC values, representing minimal important deterioration, ranged from 0.4 to 2.5 T-score points for the other domains.

**Conclusion:**

We found sufficient responsiveness of PROMIS CATs Physical Function, Fatigue, Sleep Disturbance, and Depression. The results for PROMIS CATs Pain Interference were almost sufficient, but some results for Anxiety and Ability to Participate in Social Roles and Activities were not as expected. Reported MIC values should be interpreted with caution because most patients did not change.

## Background

In the Netherlands, the Dialysis Symptom Index (DSI) and the generic 12-item Short-Form health survey (SF-12) are routine used in daily clinical care for patients with chronic kidney disease (CKD) [1]. However, the Patient-Reported Outcomes Measurement Information System (PROMIS®) was recently selected as preferred generic instruments for use in daily medical specialty care across conditions by a national working group of representatives of all umbrella organizations involved in Dutch medical specialist care together with PROM experts and patient organizations, under the auspices of the Dutch Ministry of Health, Welfare, and Sport (program “Outcomes Based Healthcare”) [2]. Internationally, a combination of PROMIS Global Health and PROMIS-29 has been recommended as one of three possible PROMs for use in patients with chronic kidney disease (CKD) by a consensus group of stakeholders of the International Consortium of Health Outcomes Measurement (ICHOM) [3].

PROMIS is a generic system of highly efficient, extensively validated patient-reported outcome measures (PROMs) that can be used to measure commonly relevant aspects of health-related quality of life (HRQOL), such as fatigue, anxiety, physical function, and social participation, in people with and without (chronic) medical conditions [4]. PROMIS consists of a collection of item banks. An item bank is a large set of questions that measure one domain (e.g. physical function). Item banks were developed using item response theory (IRT) modelling, and can be administered either as fixed short forms or as a computerized adaptive test (CAT). In a CAT, the computer selects questions from the item bank based on the answers to previous questions. The CAT is adapted to the symptom severity or functional level of the patient, resulting in questions that are likely more relevant to the patient. In addition, on average less questions are required to obtain similar or even more precise measurements compared to fixed PROMs measuring similar domains [5; 6]. Sufficient validity and reliability of PROMIS short forms and CAT was found in U.S. patients with CKD [7–9]. In a recent study we also found sufficient construct validity and test-retest reliability of seven PROMIS CATs (assessing physical function, pain interference, fatigue, sleep disturbance, anxiety, depression, and ability to participate in social roles and activities) in Dutch patients with advanced CKD [10]. However, responsiveness of PROMIS has not yet been studied in patients with CKD.

The recommendations for PROMIS in the Netherlands and abroad led to the desire to validate PROMIS in CKD patients and to compare the measurement properties of PROMIS to the SF-12. In a previous study, we found better reliability and smaller measurement error of PROMIS CATs compared to the SF-12, although PROMIS CATs required six to seven items per domain (45 items in total, using a high precision stopping rule of r = 0.95) as compared to 12 items for the SF-12. Seven CATs could be completed in on average 10.2 min as compared to 3.3 min for the SF-12. The aim of the current study was to assess responsiveness and minimal important change (MIC) of seven PROMIS CATs (Physical Function, Pain Interference, Fatigue, Sleep Disturbance, Anxiety, Depression, and Ability to Participate in Social Roles and Activities) in patients with advanced CKD, using 6 months follow-up data of this previous study [10].

## Methods

### Study design

A longitudinal study was performed, in which, after providing written informed consent, patients with advanced CKD were invited by e-mail to complete the PROMs digitally at the KLIK research platform (www.hetklikt.nu) at 3 time points; at inclusion (i.e. baseline), after 2 weeks (for assessing test-retest reliability, as described in a separate paper [10]) and after 6 months. We used baseline and 6 months measurements for this study. During follow-up patients received care as usual, which could include starting hemodialysis, peritoneal dialysis, or transplantation. The study was designed and reported according to COSMIN guidelines [11; 12]. A sample size of 100 was considered “very good” according to COSMIN [11].

### Participants

We included adult patients with advanced CKD with an estimated glomerular filtration rate (eGFR) < 30 ml/min.1.73m^2^, not receiving dialysis treatment. Exclusion criteria were start with kidney replacement therapy (KRT; dialysis or kidney transplantation) planned within 4 weeks after inclusion, rapid deterioration of kidney function (i.e. decrease in eGFR of > 20 ml/min.1.73 m^2^ during the last 6 months before inclusion), not able to complete questionnaires due to cognitive impairment, poor knowledge of the Dutch language, or no informed consent. Patients were recruited between November 2020 and August 2021 by their nephrologist at the outpatient clinics of Amsterdam UMC and “Niercentrum aan de Amstel” in Amstelveen, the Netherlands [10]. The study population represents the population in which PROMs are being used in these centers. Eligible patients received written information by mail and were, if needed, approached by telephone after 2 weeks for further information. Patients without access to an electronic device with internet connection could participate by telephone.

### Measures

We collected the following information from the medical records of the participants: age, gender, primary kidney disease according to European Renal Association codes [13], body mass index (BMI, weight (kg)/height (m)^2^), smoking status, comorbidities (hypertension, diabetes mellitus, cardiovascular disease, lung disease, liver disease and malignancy) as defined by ICHOM [3], eGFR (ml/min/1.73m^2^, calculated with the CKD-EPI equation [14]) at each time point, kidney replacement therapy (KRT) in medical history, start of KRT during follow-up and death during follow-up. Patients reported educational level and ethno-cultural background at baseline.

Participants completed the following PROMs at baseline and at six months follow-up through the KLIK research platform [15], which is a PROM platform connected to the CAT software of the Dutch-Flemish Assessment Center, part of the Dutch-Flemish PROMIS National Center [16]:

- Seven Dutch-Flemish PROMIS CATs [17]: v1.2 Physical Function, v1.1 Pain Interference, v1.0 Fatigue, v1.0 Sleep Disturbance, v1.0 Anxiety, v1.0 Depression, and v2.0 Ability to Participate in Social Roles and Activities. All items have five response options (e.g. ranging from ‘never’ to ‘always’ or from ‘not at all’ to ‘very much’). In this study, the CAT stopped when a SE of 2.2 on the T-score metric was reached (comparable to a reliability of approximately 0.95) or when a maximum of 12 items per CAT was administered. We used a lower SE compared to the standard stopping rule (i.e. SE: 3.0)[5] because a higher reliability may be preferable for routine care and by using this setting, the optimal performance of PROMIS CATs could be investigated. PROMIS CAT scores were calculated based on the original US item parameters, as per PROMIS convention, and are expressed as T-scores where a score of 50 represents the average score of the U.S. general population, with a SD of 10. Higher scores indicate more of the construct (e.g. a higher score for Depression means more depressive symptoms, a higher score for Physical Function means more [better] physical functioning). In addition, for comparison with the SF-12 component summary scores, we calculated the PROMIS-29 physical and mental health summary scores [18]. Finally, for descriptive purposes only, we also calculated the PROMIS-Preference (PROPr) score, which provides a preference-based summary score (health utility) for economic evaluations. The PROPs score was calculated according to the prediction model described by DeWitt et al., using preferences from the US population [19].
- PROMIS item v1.0 Numerical Rating Scale Pain Intensity 1a, a single item with a 0–10 scale, with higher scores indicating more pain.
- 12-item Short-Form health survey (SF-12) version 2 [21; 22], a generic PROM assessing the following aspects of HRQOL: physical functioning, role-physical, bodily pain, general health, vitality, social functioning, role-emotional and mental health. To enable comparison with PROMIS domains, we calculated eight domain scores (not part of the official SF-12 scoring). For physical functioning, physical and emotional role functioning, and mental health the two available items were summarized. For bodily pain, vitality, social functioning, and general health single items were used. Additionally, we calculated the overall physical component summary (PCS) score and the mental component summary (MCS) score based on weighted summaries of all items, using the standard SF-12 scoring algorithm. Domain scores were transformed to a score from 0 to 100, while the PCS and PCS scores have a mean of 50, representing the average score of the U.S. general population, with a SD of 10. Higher scores indicate better HRQOL. The SF-12 showed sufficient validity in patients with CKD [22–24] and is routinely used in Dutch nephrology care [25].
- Dialysis Symptom Index (DSI) [26], a 30-item kidney disease specific PROM to assess physical and emotional symptom burden. Patients report the presence of 30 symptoms (yes/no) during the past week and, if present, the burden of each symptom on a 5-point polytomous response scale ranging from 1 ‘not at all’ to 5 ‘very much’ bothersome. We calculated two sum scores: (1) total number of symptoms present (0–30 symptoms), and (2) total symptom burden score, which is the sum of burden on individual symptoms ranging from 0 (no symptoms) to 150 (all 30 symptoms are present and very much bothersome) [26; 28]. The DSI items ‘feeling tired or lack of energy’, ‘feeling anxious’, ‘trouble falling asleep’ and ‘trouble staying asleep’ (the latter two items are hereafter combined as ‘sleep problems’) were used as comparison items in the responsiveness analyses of the PROMIS CATs since these items intend to measure constructs comparable to the PROMIS CAT domains Fatigue, Anxiety and Sleep Disturbance, respectively. The DSI showed sufficient validity in patients with CKD [26] and is routinely used in Dutch nephrology care [25].
- At six months follow-up patients were also asked to rate their perceived change in each of the seven PROMIS domains on a Global Rating Scale (GRS) (e.g. `How did your fatigue change compared to 6 months ago?`). Perceived change was rated on a five-point scale (much worse, a little worse, no change, a little better, much better).

The PROMs (seven PROMIS CATs, SF-12 and DSI) were presented in random order across patients, but with fixed order within patients during follow-up (i.e. an individual patient received the measures in the same order each time but the order differed from patient to patient). The KLIK platform did not allow for any missing values within questionnaires.

### Responsiveness

Responsiveness of the PROMIS CATs was determined by comparing changes in PROMIS CAT T-scores to changes in scores of the PROMIS Pain Intensity, SF-12 (MCS, PCS and separate domains), and DSI (items/domains and overall), and to the GRS. On average, we expected that patients with advanced CKD would slightly deteriorate in physical functioning and participation and would not much change in mental functioning over a period of 6 months [29; 30]. Therefore, we expected relatively low correlations. However, we expected that there would be at least some variation in outcomes and that some patients would improve and some patients would deteriorate and that this variation would be sufficient to evaluate the responsiveness of the PROMIS CATs [30]. To support the responsiveness of PROMIS CAT, we hypothesized that the correlations between changes in PROMIS CAT T-scores and changes in comparable domains of the comparator instruments would be at least 0.40 (rather than 0.50 suggested by COSMIN) [31]. Furthermore, we hypothesized that per comparator instrument, the correlations between changes in PROMIS CAT T-scores and changes in the comparator instrument should be the highest for comparable domains (Table 1) [31]. Although the aim of the study was not to validate the PROMIS-29 summary scores, we expected that the correlations between the PROMIS-29 summary scores and the SF-12 component scores would be at least 0.40. We also expected that changes in all PROMIS CAT T-scores would be related to changes in the total number of symptoms and changes in symptom severity, as measured with the DSI. We expected that these correlations would be higher than the correlations with other DSI scores but lower than the correlations with changes in similar domains of the SF-12. We also calculated effect sizes for all PROM scores (defined as mean change divided by baseline standard deviation) and we expected at least similar or slightly higher effect sizes for PROMIS CAT compared to comparable SF-12 domains and DSI items. Finally, we examined the ability of the PROMIS CATs to distinguish between patients who reported to be deteriorated (a little worse or much worse on the GRS) and patients who reported to be not deteriorated (no change, a little better, much better on the GRS). The area under the Receiver Operating Characteristics (ROC) Curve (AUC) was used as a measure of responsiveness. An AUC of at least 0.70 is generally considered sufficient evidence for responsiveness [31]. We considered responsiveness sufficient if at least 75% of the results were in accordance with the hypotheses.

**Table 1:** Expected and observed correlations between PROMIS CAT change scores and change scores in SF-12 and DSI

	PROMIS Physical Function	PROMIS Pain Interference	PROMIS Fatigue	PROMIS Sleep Disturbance	PROMIS Anxiety	PROMIS Depression	PROMIS Ability to Participate
**PROMIS single item**							
Pain Intensity (0–10)	-0.22	0.58*	0.28	0.09	0.10	0.14	-0.26
**SF-12**							
Physical functioning	0.39*	-0.26	-0.21	0.00	-0.12	-0.12	0.22
Role-physical	**0.44**	-0.25	-0.23	-0.17	-0.10	-0.20	0.36*
Bodily pain	0.19	-0.55*	-0.16	-0.06	-0.17	-0.21	0.15
General health	0.31	-0.16	-0.35	-0.11	-0.11	-0.21	0.24
Vitality	0.17	-0.11	-0.41*	-0.10	-0.10	-0.08	0.29
Social functioning	0.22	-0.23	-0.20	-0.06	-0.18	-0.17	0.41*
Role-emotional	0.18	-0.13	-0.12	-0.13	**-0.36**	**-0.36**	0.34*
Mental health	0.14	-0.18	-0.18	-0.20	-0.30*	-0.43*	0.27
Physical component summary^*^	**0.47**	-0.45	-0.31	-0.05	-0.04	-0.09	0.24
Mental component summary^*^	0.11	-0.10	-0.21	-0.18	-0.37*	-0.40*	0.42
**DSI**							
Number of symptoms (0–30)	*-0.35*	*0.23*	*0.37*	*0.29*	*0.25*	*0.23*	*-0.34*
Symptom burden score (0-150)	*-0.36*	*0.27*	*0.38*	*0.31*	*0.26*	*0.26*	*-0.33*
Feeling tired or lack of energy (0–5)	-0.36	0.18	0.41*	0.03	0.11	0.15	-0.20
Sleep problems (0–10)^#^	-0.15	0.02	0.14	0.57*	0.02	0.01	-0.20
Feeling anxious (0–5)	-0.06	0.15	0.08	0.12	0.42*	0.29	-0.19
**Global Rating Scale**							
Change in physical function	0.45*	0.25	0.34	0.18	0.11	0.17	0.26
Change in pain	0.29	0.28*	0.27	0.06	0.12	0.11	0.19
Change in fatigue	0.38	0.18	0.37*	0.10	0.03	0.09	0.22
Change in sleep disturbance	0.32	0.12	0.32	0.37*	0.01	0.04	0.14
Change in anxiety	0.24	0.15	0.29	0.24	0.04*	0.11	0.02
Change in depression	0.17	0.13	0.25	0.17	0.05	0.20*	0.07
Change in ability to participate	0.38	0.20	0.32	0.17	0.13	0.22	0.32*

* Expected correlations of at least 0.40

Bold = Per comparator instrument, the PROMIS CAT was expected to have the highest correlations with scales measuring similar domains

Italic = the PROMIS CATs were expected to have higher correlations with these DSI domains than with the other DSI domains (but lower than the bold correlations with similar domains of the other comparator instruments)

^*^ SF-12 physical component summary includes the domains physical functioning, role-physical, bodily pain and general health; SF-12 mental component summary includes the domains vitality, social functioning, role-emotional and mental health

^#^ DSI Sleep problems were defined as trouble falling asleep and/or trouble staying asleep

Abbreviations: PROMIS, Patient-Reported Outcomes Measurement Information System; CAT, Computerized Adaptive Test; SF-12, 12-item Short Form Health Survey; DSI, Dialysis Symptom Index

### Minimal important change

MIC was defined as the smallest change in score that patients consider important [32]. Because patients were expected to deteriorate, a minimal important deterioration was calculated instead of a minimal important improvement. A prerequisite for calculating the MIC was a correlation between the PROMIS CAT change score and the GRS of at least 0.30 [33]. The MIC was estimated using predictive modelling, where the MIC was defined as the change score where the post-test probability of belonging to the deteriorated group equals the pre-test probability (i.e. the proportion deteriorated patients) [34]. Terluin et al. showed that the predictive modelling approach is more precise than the commonly used ROC method [34] and that ROC MIC values are biased when the percentage of deteriorated (or improved) patients is not 50% [35]. The predictive modelling approach can correct for this. MIC values were therefore adjusted for the high proportion of deteriorated patients [35], and bootstrapping was used to obtain confidence intervals.

## Results

### Participants

Almost half of the patients that were approached by their nephrologist provided written informed consent (for details, see [10]). In total, 207 participants completed the baseline measurement and 186 (90%) participants completed the 6 months follow-up. Characteristics of the study population are shown in Table 2. Mean (SD) age was 65.5 (13.8) and 60% were male. Mean (SD) eGRF at baseline was 21.4 (6.7). Mean (SD) eGRF at follow-up was 22.9 (10.5). During follow-up, 12 patients died, six patients started hemodialysis, one patient started peritoneal dialysis, and six patients were transplanted.

**Table 2:** Characteristics of the study population at baseline (n = 207)

	n (%) or mean (SD)
Sex, male	124 (59.9)
Age, years	65.5 (13.8)
Ethno-cultural group^$^, Dutch	176 (85.0)
Educational level^#^	
Low	85 (41.0)
Middle	49 (23.7)
High	73 (35.3)
Primary kidney disease	
Glomerulonephritis	34 (16.6)
Pyelonephritis	7 (3.4)
Polycystic kidney disease	16 (7.8)
Other congenital/hereditary kidney diseases	15 (7.3)
Hypertension/renal vascular disease	46 (22.5)
Diabetes mellitus	14 (6.8)
Miscellaneous	63 (30.7)
Unknown	10 (4.9)
Kidney function, eGFR (ml/min.1.73 m^2^)	21.4 (6.7)
KRT in medical history^£^, yes	35 (17.0)
BMI, kg/m^2^	26.8 (5.2)
Smoking	
Yes	25 (13.2)
No, stopped	94 (49.7)
No, never smoked	70 (37.0)
Comorbidities	
Hypertension, yes	164 (79.2)
Diabetes mellitus, yes	62 (30.0)
Cardiovascular disease, yes	53 (25.6)
Lung disease, yes	30 (14.5)
Liver disease, yes	11 ( 5.3)
Malignancy, yes	50 (24.2)

^$^ Self-reported ethno-cultural group: “What ethnic group do you consider yourself to belong to?“

^#^ Educational level according to International Standard Classification of Education (ISCED) levels 2011, classified as low: primary, lower secondary or lower vocational education; middle: upper secondary or upper vocational education; high: tertiary education (college/university)

^£^ KRT in medical history includes patients who have undergone (temporary) dialysis treatment or had received a kidney transplant in the past. At study inclusion, all patients had an eGFR < 30 and did not require dialysis treatment, in accordance with inclusion criteria

Missing values for population at baseline: primary kidney disease: n = 2 (1.0%); KRT in medical history: n = 1 (0.5%); BMI: n = 11 (5.3%); smoking: n = 18 (8.7%)

Abbreviations: BMI = body mass index; eGFR = estimated glomerular filtration rate; KRT = kidney replacement therapy

Scores and changes in scores of PROMIS CATs and other PROMs at baseline and at 6 month follow-up are presented in Table 3. Patients with advanced CKD had lower physical function (43.6) and higher pain interference (51.9) and fatigue (53.2) than the average population values of 50, while scores for the other domains were closer to 50. Mean changes in scores after 6 months were very small for all PROMs (≤ 1.1 T-score point for PROMIS CATs and < 1 point for SF-12 domains).

**Table 3:** Mean(SD) PROM (change) scores at baseline and 6 months follow-up and effect sizes

	Baseline N = 207	Follow-up N = 186	Change N = 186	Effect size*
**PROMIS CAT**				
Physical Function	43.4 (8.3)	43.0 (8.0)	-0.9 (4.6)	-0.11
Pain Interference	51.9 (9.1)	51.6 (9.2)	0.1 (7.4)	0.01
Fatigue	53.2 (8.7)	53.0 (9.4)	0.0 (6.5)	0.00
Sleep Disturbance	49.3 (7.9)	49.4 (8.4)	0.0 (5.4)	0.00
Anxiety	51.2 (7.7)	50.8 (8.0)	-0.2 (5.7)	0.03
Depression	49.8 (7.5)	49.6 (8.0)	0.0 (5.7)	0.00
Ability to Participate in Social Roles and Activities	49.2 (8.6)	48.4 (9.1)	-1.1 (7.3)	-0.13
**PROMIS single item**				
Pain Intensity (0–10)	1 (0–5)	2 (1–6)	0 (-1–1)	0.00
**PROMIS-29 physical health summary score**	44.0 (8.4)	43.6 (8.3)	-0.9 (4.7)	-0.11
**PROMIS-29 mental health summary score**	48.6 (7.3)	48.5 (8.2)	-0.3 (4.8)	-0.04
**PROPr**	0.40 (0.19)	0.39 (0.20)	-0.01 (0.11)	-0.06
**SF-12**				
Physical functioning	40.5 (11.3)	41.1 (11.2)	-0.1 (9.4)	0.01
Role-physical	40.1 (10.3)	41.2 (10.0)	0.8 (9.3)	0.08
Bodily pain	46.9 (11.3)	47.8 (11.4)	0.6 (11.1)	0.05
General health	36.3 (10.9)	36.5 (11.1)	-0.2 (9.3)	-0.02
Vitality	48.5 (10.2)	47.9 (10.6)	-0.9 (8.8)	-0.09
Social functioning	43.4 (12.1)	43.0 (11.4)	-0.9 (12.0)	-0.07
Role-emotional	44.2 (11.3)	44.1 (10.8)	-0.5 (12.5)	-0.04
Mental health	50.1 (9.3)	50.7 (9.3)	0.0 (8.0)	0.00
Physical component summary^*^	39.2 (10.7)	40.0 (10.5)	0.4 (7.9)	0.04
Mental component summary^*^	49.3 (9.7)	48.9 (9.2)	-0.8 (8.9)	-0.08
**DSI**				
Number of symptoms (0–30)	9.4 (5.6)	8.8 (5.8)	-0.4 (4.6)	-0.07
Symptom burden score (0-150)	22 (12–36)	20 (10–33)	0.0 (-7–6)	0.0
Feeling tired or lack of energy (0–5)^^^	2.0 (1.6)	2.0 (1.6)	0.0 (-0.3-1.0)	0.0
Sleep problems (0–10)^^#^	2.0 (0–3)	2 (0–4)	0 (-1–1)	0
Feeling anxious (0–5)^^^	0 (0–0)	0 (0–0)	0 (0–0)	0

^*^ SF-12 physical component summary includes the domains physical functioning, role-physical, bodily pain and general health; SF-12 mental component summary includes the domains vitality, social functioning, role-emotional and mental health

^^^ Prevalence of feeling tired or lack of energy: 70.0%, sleep problems: 52.7%, feeling anxious: 18.7%

^#^ Sleep problems were defined as trouble falling asleep and/or trouble staying asleep

Abbreviations: PROMIS, Patient-Reported Outcomes Measurement Information System; CAT, Computerized Adaptive Test; SF-12, 12-item Short Form Health Survey; DSI, Dialysis Symptom Index; SD, standard deviation; IQR, interquartile range

* Effect size = mean change / SDbaseline

### Responsiveness

Correlations between changes in PROMIS CAT T-scores and changes in SF-12, changes in DSI scores, and GRS scores are presented in Table 4. Effect sizes are presented in Table 3. Table 5 provides an overview of how many hypotheses were confirmed. For PROMIS CAT Physical Function, Fatigue, Sleep Disturbance, and Depression sufficient responsiveness was found as more than 75% of the results were in accordance with the hypotheses. For PROMIS CAT Pain Interference and Anxiety, 70% and 60% of the results were in accordance with the hypotheses. For PROMIS CAT Ability to Participate in Social Roles and Activities only 42% of the results were in accordance with the hypotheses. As expected, the correlations between the PROMIS-29 summary scores and the SF-12 component scores were higher than 0.40 (0.52 for the physical scores, and 0.44 for the mental scores, respectively).

**Table 4:** Area under the ROC curve (AUC), representing the ability of PROMIS CATs to distinguish patients who deteriorated from patients who did not deteriorate, and minimal important change (MIC), representing a minimal important deterioration

	AUC	MIC (95%CI)
**PROMIS CAT**		
Physical Function	0.71	-1.6 (-3.2-0.2)
Pain Interference	0.67	1.6 (-8.1-9.2)
Fatigue	0.71	0.4 (-2.8-3.4)
Sleep Disturbance	0.75	1.1 (-1.8-3.5)
Anxiety	0.52	*
Depression	0.76	*
Ability to Participate in Social Roles and Activities	0.67	-2.5 (-6.5-2.1)

* Because the correlation between the PROMIS change score and the global rating of change was too low, these MIC values were not calculated

**Table 5:** Number of responsiveness hypotheses confirmed

	Correlation ≥ 0.40 with similar domains	Highest correlation with similar SF-12 domains	Highest correlation with similar DSI domains	Higher correlation with DSI total number of symptoms and changes in symptom severity than with other DSI scores	At least similar or slightly higher effect sizes for PROMIS CAT compared to comparable SF-12 domains	At least similar or slightly higher effect sizes for PROMIS CAT compared to comparable DSI items	AUC ≥ 0.70	Total number of hypotheses confirmed (%)
**PROMIS CAT**								
Physical Function	2/2	3/3	NA	1/2	3/3	NA	1/1	10/11(91)
Pain Interference	2/3	2/2*	NA	2/2	1/2*	NA	0/1	7/10 (70)
Fatigue	2/3	1/1	1/1	2/2	0/1	1/1	1/1	8/10 (80)
Sleep Disturbance	1/2	NA	1/1	2/2	NA	1/1	1/1	6/7 (86)
Anxiety	1/4	3/3	1/1	2/2	1/3	1/1	0/1	9/15 (60)
Depression	2/3	3/3	NA	2/2	1/3	NA	1/1	9/12 (75)
Ability to Participate in Social Roles and Activities	1/4	0/3	NA	2/2	2/2	NA	0/1	5/12 (42)

* Including expected high correlation with (change in) PROMIS Pain Intensity

Supplementary Table 1. Mean (SD) change in PROMIS CAT T-scores across levels of self-rated change in the same construct

### Minimal important change

Supplementary Table 1 presents changes in PROMIS CAT T-scores across all categories of the GRS. Because the much improved and much worse groups were small, the means were not always monotonically ordered, although for most domains the mean changes were largest in the much improved and much worse groups, as expected. Because the correlations between the PROMIS CAT change scores and the GRS were much lower than 0.30 for Anxiety and Depression, a MIC for these domains was not calculated. The MIC representing minimal important deterioration was − 1.6 T-score points for PROMIS Physical Function, 1.6 for Pain Interference, 0.4 for Fatigue, 1.1 for Sleep Disturbance, and − 2.5 for the Ability to Participate in Social Roles and Activities.

## Discussion

The aim of this study was to assess responsiveness and minimal important change (MIC) of seven PROMIS CATs in patients with advanced CKD, measuring physical function, pain interference, fatigue, sleep disturbances, anxiety, depression, and the ability to participate in social roles and activities. On average, we expected that patients with advanced CKD would slightly deteriorate in physical functioning and participation and would not much change in mental functioning over a period of 6 months. This was indeed reflected in the changes in PROMIS scores. The pattern of correlations between change scores supported the responsiveness of the PROMIS CATs for Physical Function, Fatigue, Sleep Disturbance, and Depression, almost for Pain Interference but not for Anxiety and Ability to Participate in Social Roles and Activities. MIC values, representing the minimal important deterioration, ranged from 0.4 to 2.5 T-score points for the PROMIS CATs, except for Anxiety and Depression, for which MIC values could not be estimated.

For PROMIS CAT Ability to Participate in Social Roles and Activities only 42% of the results were in accordance with the predefined hypotheses for responsiveness. This was due to lower than expected correlations between change in PROMIS Ability to Participate and change in SF-12 Physical and Emotional role functioning (0.36 and 0.34, rather than ≥ 0.40) and a higher than expected correlation between change in PROMIS Ability to Participate and change in SF-12 MCS (0.42). We expected this latter correlation to be lower than the correlations with change in SF-12 Role-physical (0.36), Social functioning (0.41), and Role-emotional (0.34). A possible explanation could be that these SF-12 domain scores are based on one or two items only, which makes the correlations difficult to estimate The effect size of the PROMIS Ability to Participate CAT was the highest of all PROMIS CATs, so it may be too strict to argue that this PROMIS CAT is not responsive. For PROMIS Anxiety and PROMIS Depression the correlation with the GRS were also lower than expected (0.04 and 0.20, respectively). We do not have an explanation why some of the correlations were lower than expected. Perhaps response shift (i.e. a change in how patients experience their health because they adapted to their disease) or the fact that many patients did not change, played a role, but chance can also not be ruled out because we calculated many correlations. Also, predefining the magnitude of expected correlations is challenging.

This is the first study examining the responsiveness of PROMIS measures in patients with CKD. The responsiveness of PROMIS measures has been studied in patients with other chronic conditions, such as multiple sclerosis [36], COPD [37], chronic low back pain [38], and rheumatoid arthritis [39]. These studies also reported low changes in PROMIS (and other PROM) scores, because of relatively short follow-up periods, during which most patients did not change. This is an important challenge in studies assessing responsiveness in patients with chronic conditions and a limitation of this study because the aim of a responsiveness study is to detect change and patients with chronic conditions may not change much during the relatively short period of a study [32]. A longer follow-up period may lead to more variation in change scores and subsequently higher correlations between change scores.

Indirect evidence for responsiveness of PROMIS CATs was found in other studies. Sufficient construct validity and test-retest reliability was found in multiple studies in CKD patients [8–10; 41; 42]. In theory, this does not guarantee sufficient responsiveness as responsiveness may be limited due to floor or ceiling effects, but floor and ceiling effects are seldom found for PROMIS CAT, because of the large underlying item banks [42–44]. Therefore, these previous studies also support the responsiveness of PROMIS CATs in CKD patients, at least to some extent.

For PROMIS Anxiety and PROMIS Depression the correlation with the GRS were too low to calculate a MIC. This was probably due to the high proportion of patients who reported no change in anxiety (74.6%) or depression (69.7%). The estimated MIC values in this study were relatively low (0.4–2.5 T-score points) and confidence intervals were wide. A MIC value of 0.4 (representing an effect size of 0.04 of the T-score metric) for PROMIS Fatigue might be considered implausible because such a small change may not even be noticeable by patients. As stated above, the study design was not optimal because most patients did not change. Therefore, these MIC values should be interpreted with caution. A recent systematic review of PROMIS MIC values suggested that MIC values of 2–6 T-score points are reasonable to assume for PROMIS measures [45]. However, most studies included in the systematic review estimated minimal important improvement, while we estimated minimal important deterioration. Some studies also found lower MIC values for deterioration than for improvement [47; 48] but others did not find different MIC values [49; 50]. Therefore, it remains important to estimate MIC values separately for improvement and deterioration and studies with longer follow-up are needed to estimate MIC values in patients with chronic conditions.

Using PROMs in clinical practice can support the delivery of person-centered care through shared decision-making and management in CKD patients [50]. PROMIS has been recommended in national and international initiatives [2; 3]. Although the responsiveness of some of the PROMIS CATs was not sufficiently convincing, considering the evidence that we found, in combination with evidence from previous studies on construct validity, test-retest reliability, and a content comparison with the SF-12 [10], as well as the psychometric evidence and widespread implementation of PROMIS in other fields [51], we recommend the use of PROMIS in clinical practice. Reference scores of the PROMIS domains from the Dutch general population are available for all domains included in this study [52–56]. Graphical PROMIS feedback has been developed to facilitate conversations with patients [57]. PROMIS CATs are available in several electronic PROM platforms and some electronic health records (e.g. Epic) and implementation guides and training sources are available on the HealthMeasures website [58]. Administering seven PROMIS CATs takes more time than completing the SF-12 but seven PROMIS CATs can be administered within 10 min on average. An advantage of PROMIS is that each domain can be measured with a separate instrument, which provides flexibility to choose which domains to measure in studies or clinical applications. PROMIS CAT requires access to a computer and internet, which may be a limitation for some people currently. In our study, 11 patients (5%) participated by telephone. However, computer and internet use is rapidly increasing. Electronic PROM systems may also assist with remote monitoring of symptoms and functions and may encourage patients to become more engaged with their care [59]. If CAT software is not available, PROMIS short forms can be used. Although short forms may perform slightly less good than CATs, they are widely used and available in more than 60 languages and scores are directly comparable to CAT scores [59]. Healthcare providers and patients need to decide which PROMs are most relevant and feasible to use. Although there is some overlap in content with the PROMIS CATs, the DSI might be of additional value because it measures disease-specific symptoms which are not covered by PROMIS.

## Conclusion

We found sufficient responsiveness of the PROMIS CATs Physical Function, Fatigue, Sleep Disturbance, and Depression. The results for PROMIS CATs Pain Interference were almost sufficient, but some of the results for the Anxiety and Ability to Participate in Social Roles and Activities were not in line with predefined hypotheses. MIC values, representing the minimal important deterioration, ranged from 0.4 to 2.5 T-score points, but should be interpreted with caution because most patients did not change during the follow-up of this study.

## Data Availability

The data used for this research is available upon request. Contact information: Caroline B. Terwee, cb.terwee@amsterdamumc.nl.

## Abbreviations

AUC: Area under the ROC Curve
CAT: Computerized Adaptive Test
CKD: Chronic Kidney Disease
DSI: Dialysis Symptom Index
GRS: Global Rating Scale
HRQL: Health-Related Quality of Life
IRT: Item Response Theory
MIC: Minimal Important Change
PROMIS: Patient-Reported Outcomes Measurement Information System
ROC curve: Receiver Operating Characteristics Curve
SF-12: Short Form Health Survey 12
